# Supplementary material for: Interferon-β induction heterogeneity during KSHV infection is correlated to levels and activation of the transcription factors ATF2 and RelA, and not IRF3
Source: PLoS Pathog. 2026 Feb 11;22(2):e1013947. doi: 10.1371/journal.ppat.1013947 (PMC12919925; doi:10.1371/journal.ppat.1013947)
Supplement: S1 Table — (DOCX) [file ppat.1013947.s006.docx]

| Target | | Forward 5’ 🡪 3’ | Reverse 5’ 🡪 3’ | ref |
| --- | --- | --- | --- | --- |
| Human IFN-β | RNA | GTCAGAGTGGAAATCCTAAG | ACAGCATCTGCTGGTTGAAG | (84) |
| Human 18S | RNA | GTAACCCGTTGAACCCCATT | CCATCCAATCGGTAGTAGCG | (85) |
| Human MxA | RNA | ATCCTGGGATTTTGGGGCTT | CCGCTTGTCGCTGGTGTCG | (86) |
| tdTomato | RNA | ACATCCCCGATTACAAGAAGC | TTGTAGATCAGCGTGCCGTC | (87) |
| Human IFN-λ1 | RNA | CGCCTTGGAAGAGTCACTCA | GAAGCCTCAGGTCCCAATTC | (88) |
| Human ATF3 | RNA | CAGTCACTGTCAGCGACAGACCC | TCTTCTTCAGGGGCTACCTCGG | (89) |
| Human GADD45B | RNA | CGAGTCGGCCAAGTTGATGA | GGATTTGCAGGGCGATGTCA | This study |
| Human HSPA1A | RNA | GCCTTTCCAAGATTGCTGTT | TCAACATTGCAAACACAGGA | (90) |
| Human IL-6 | RNA | GGTACATCCTCGACGGCATCT | GTGCCTCTTTGCTGCTTTCAC | (91) |
| Human TNFAIP3 | RNA | GCTGCTGCCTCAGGGAAAGTC | CTCTTCTGTCCTTTTGGCCTC | (92) |
| KSHV ORFK5 | RNA | GGCGTGTACGACACGAGTGA | GCGTACTGCTTGCCACGTT | (93) |
| KSHV ORF50 | RNA | ACCAAGGTGTGCCGTGTAGAGATT | AGCCTTACGCTTCTTTGAGCTCCT | (94) |
| KSHV ORF57 | RNA | GGTGTGTCTGACGCCGTAAAG | CCTGTCCGTAAACACCTCCG | (95) |
| KSHV ORF59 | RNA | CTCCCTCGGCAGACACAGAT | GCGTGGTGCACACCGACGCCC | (96) |
| KSHV ORF42 | RNA | GAAGACATGGTGACCCACATAG | CGTCCTCTCATGTGTCTTTCTG | (97) |
| KSHV ORF52 | RNA | AAATCGAAGCCAGGGTCAGG | CTCCTCTTCGTCGCCTGTTATTG | (95) |
| Human CCR5 | DNA | ATGATTCCTGGGAGAGACGC | AGCCAGGACGGTCACCTT | (98) |
| Human mtDNA | DNA | GCCACAGCACTTAAACACATCTCT | TGAAATCTGGTTAGGCTGGTGTTAG | (99) |

S1 Table. Primers used for qPCR in this study.
