# Supplementary material for: Interferon-β induction heterogeneity during KSHV infection is correlated to levels and activation of the transcription factors ATF2 and RelA, and not IRF3
Source: PLoS Pathog. 2026 Feb 11;22(2):e1013947. doi: 10.1371/journal.ppat.1013947 (PMC12919925; doi:10.1371/journal.ppat.1013947)
Supplement: Attachment — Submitted filename: revision_plan_PPath_final.pdf [file ppat.1013947.s007.pdf]

Revision Plan

Review COMMONS

Manuscript number: RC-2025-03136
Corresponding author(s): Marta Maria, Gaglia

1. General Statements

We thank the reviewers for recognizing the importance of study, and how it "addresses a long-standing question in the heterogeneity of cellular responses to stressors", "makes a conceptual advance by identifying transcription factors as the limiting determinant of IFN-β induction in KSHV-infected cells", and "serves as a crucial starting point for understanding cellular heterogeneity". We agree that our findings appeal to a broad audience interested in virology and immunology.

We also thank the reviewers for their insightful suggestions that will greatly strengthen our study. Below we detail how we plan to address their comments experimentally and how we have already edited the text to respond to them.

2. Description of the planned revisions

Reviewer #2

1) While the study presents intriguing evidence for AP-1 involvement in regulating IFN-β responses, the reliance on total c-Jun levels as a readout is limiting. Because c-Jun activity depends on phosphorylation and promoter binding, additional experiments (i.e., phospho-c-Jun analysis or ChIP at the IFNB1 promoter) would strengthen the link between AP-1 activity and the observed reporter outcomes.

We agree that that a stronger link between AP-1 activity and IFN induction would improve our study. To this end, we have cloned interferon-β reporter constructs that contain mutations in either the AP-1, IRF3, or NF-κB binding sites, to mechanistically test the connection between AP-1 and activation of the IFN promoter. Also, if we are able to obtain a sufficient number of sorted cells, we will perform ChIP-PCR for IFN promoter occupancy by the transcription factors. We have previously investigated c-Jun and ATF2 phosphorylation during KSHV reactivation and caspase inhibition. Surprisingly, in preliminary experiments we did not detect phosphorylation of either AP-1 subunit. We will confirm this result and add these data to the manuscript.

2) The data presented demonstrating that Serine 386 phosphorylation does not distinguish first responder cells is strong. Including complementary data on Ser396 phosphorylation would strengthen the conclusion, as this well-established activation marker is readily detectable with available reagents and would help confirm that the potentiation of IRF3 activity is not the driver of the observed heterogeneity.

We will complement the Ser386 results with Ser396 staining.

Reviewer #3

A major worry comes from using lentiviral transduction to insert the reporter promoter into cells without selecting for clones. Lentiviral transduction introduces heterogeneity due to random insertion of their vector. This results in different copy numbers for the reporter construct, leading to heterogeneity in the reporter expression. Additionally, the expression of foreign proteins, particularly in immune cells, can be perceived as danger signals (10.1007/s12015-016-9670-8) and occasionally trigger p65 activation. To control for this, the authors could validate their reporter results by including a non-IFNb promoter (e.g., constitutive) expressing tdTomato and verifying that these cell populations do not also express endogenous IFNb mRNAs.

We did not select clonal cell lines because different cells may have different reactivation propensity. Moreover, we did not want the tdTomato signal to reflect specific regulation of a single genomic region. We have now added an explanation as to why we did not clonally select that cell lines in the Results section (line 157). Our control conditions that do not result in IFN-β induction show that lentiviral insertion is not sufficient to cause IFN induction, as we did not detect IFN-β mRNA in the untreated reporter cells (first bar in Figure 1C). We also clarified in the Results section (line 184) that the selective enrichment of both IFN-β and tdTomato mRNA in the sorted tdTomato+ cells demonstrates that tdTomato is a faithful reporter for rare IFN-β expression, regardless of heterogeneous lentiviral transduction in the population. To further verify that lentiviral transduction does not play a role in introducing heterogeneity in induction of our tdTomato reporter and of IFN-β, we will measure IFN-β levels in BC-3 cells expressing tdTomato under the control of a CMV promoter, which we have already created. In this line, not all the cells express high tdTomato levels. We will thus be able to sort based on the tdTomato signal and confirm that in this case, tdTomato expression is not predictive of IFN induction.

Regarding AP1 and NF-kB activation, the authors could investigate downstream genes such as GADD45B, HSPA1A, and ATF-3 (for AP1), and IL-6, TNFAIP3 (A20) (for both AP1 and NFkB). It would be interesting to determine if these genes are exclusively expressed in tdTomato-expressing cells.

We will quantify the mRNA levels of these genes by performing qPCR on cells sorted based on tdTomato expression. Preliminary results suggest some of these transcripts are enriched in the tdTomato (and IFN) positive cells.

While the authors observed no direct correlation between c-Jun alone and IFN-b production, it is conceivable that TPA-induced c-Jun primes the cells that become fully transcriptionally active upon a stimulus like viral reactivation. I propose that the authors attempt to inhibit c-Jun activation during KSHV reactivation (TPA + caspase inhibitor) using inhibitors like SP600125 and subsequently assess whether this blockade reduces the proportion of IFNb+ cells.

We have tried using the suggested inhibitor (SP600125), but found that it inhibits KSHV reactivation, making any result on IFN levels difficult to interpret. Currently, we are testing a dual AP-1 and NF-κB inhibitor (SP100030) and preliminary results suggest that it does not reduce reactivation, but does reduce IFN induction.

3. Description of the revisions that have already been incorporated in the transferred manuscript

We have edited some language in the manuscript to address comments made by the reviewers. These changes are highlighted in yellow in the text.

Reviewer #2

3) Consider updating the title to more directly reflect the findings (e.g. "Interferon-β induction heterogeneity during KSHV infection is correlated to expression of ATF2 and RelA")

We have updated the title to “Interferon-β induction heterogeneity during KSHV infection is correlated to levels and activation of the transcription factors ATF2 and RelA, and not IRF3”

4) To ease the interpretation of data, indicate what the black and white circles indicate in the figure legends.

We have updated the figures to be more intuitive, using + and -.

5) IE ORF50 is used to show no differences between first responders and non-responders, but showing early and late genes across tdTomato positive and negative cells would rule out potential differences in progression through reactivation.

We added a clarification in the Results section (line 195), explaining that we have examined the progression of viral reactivation through single-cell transcriptomics in our previous publication, and that the results indicate that viral gene expression plays a small role in interferon-β heterogeneity. We favor the scRNA-seq dataset for this conclusion, because the tdTomato negative cell population represents a mix of non-reactivating cells, which would not be expected to make IFN, and reactivating cells that fail to induce IFN expression.

6) The data in Figure 5D (quantified in E and F) show a compelling trend. This could be further clarified by plotting a trend line that connects the results of independent experiments, rather than only showing individual data points. Such visualization would make the consistency of the observed trend across experiments more apparent.

We have added lines in the graphs in Figure 5 to ease visualization.

4. Description of analyses that authors prefer not to carry out

Reviewer #1

One experiment that may provide some insight into the selective RelA activation is to quantify viral genomes within the high and low IFN producing cells. Perhaps, the genome as a PAMP, is more abundant in the inducing cells.

We have added a note in the Discussion section (line 417) that we have evidence that the cGAS PAMP in our system is mitochondrial DNA, not viral DNA. Moreover, our results suggest that the variation in PAMP levels are not the source of heterogeneity, as this would cause heterogeneous activation of the cGAS-STING-TBK1-IRF3 axis. Instead, we have discovered that TBK1 and IRF3 are activated even in cells without interferon-β induction.
