# Supplementary material for: Interferon-β induction heterogeneity during KSHV infection is correlated to levels and activation of the transcription factors ATF2 and RelA, and not IRF3
Source: PLoS Pathog. 2026 Feb 11;22(2):e1013947. doi: 10.1371/journal.ppat.1013947 (PMC12919925; doi:10.1371/journal.ppat.1013947)
Supplement: Attachment — Submitted filename: KakuGaglia_response_PPathresub_v5.pdf [file ppat.1013947.s008.pdf]

Response to reviewers' comments from Reviewer Commons submission (PPATHOGENS-D-25-02684)

We thank the reviewers and the PLOS Pathogens team for the support and the constructive criticism. We believe we have thoroughly addressed the comments by adding new data and revising the text and figures as suggested. We have added 5 new supplemental figures and several panels to the main figures. We have included a version of the manuscript where the altered text is highlighted in yellow. Here is a point-by-point response. Reviewers' comments are in green and italics and our responses in black. We have also embedded figures in the response for ease of review.

Reviewer #1:

One experiment that may provide some insight into the selective RelA activation is to quantify viral genomes within the high and low IFN producing cells. Perhaps, the genome as a PAMP, is more abundant in the inducing cells.

We had also considered this hypothesis when we started the project and did work to identify the PAMP/DAMP in our system. We discovered that the PAMP is mitochondrial DNA. However, in other experiments, we have discovered that TBK1 and IRF3 are activated even in cells without interferon (IFN)-β induction. This indicates that the pathway downstream of the PAMP and cGAS is activated in many, if not all cells. Therefore, the variation in PAMP levels cannot be the source of heterogeneity, as this would cause heterogeneous activation of the cGAS-STING-TBK1-IRF3 axis. We now discuss this in the Discussion section (lines 452-455). We have also added a Supplemental Figure (S3 Fig) showing that mitochondrial DNA is the PAMP and updated the text to include this information in the Results and Discussion sections (lines 313-316, 453-454).

S3 Fig

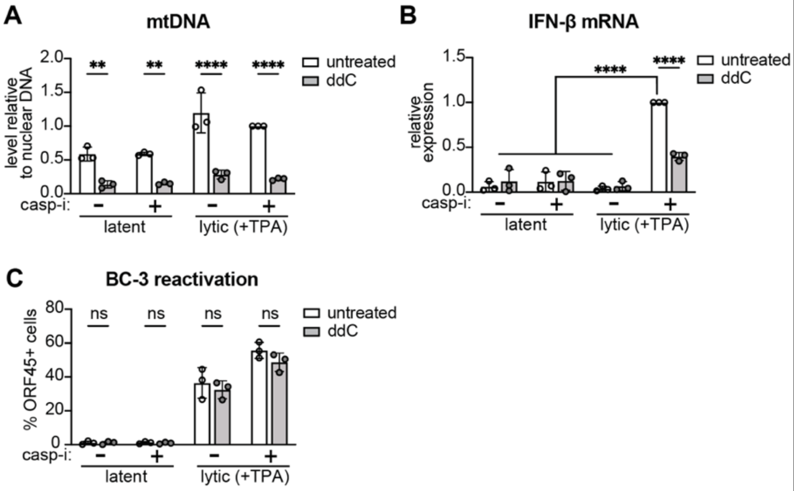

S3 Fig. Mitochondrial DNA is the likely cGAS ligand during lytic KSHV infection. BC-3 cells were treated with or without 20 μM dideoxycytidine ("ddC") for 48 hours to deplete mitochondrial DNA ("mtDNA"), then treated with 20 ng/ml TPA for 48 hours to induce the lytic cycle ("lytic (+TPA)"), or vehicle (DMSO, "latent"). At the time of lytic induction, where indicated, the cells were also treated with 10 μM of the pan-caspase inhibitor IDN-6556 ("casp-i"). **A.** DNA was extracted and mtDNA levels were measured by qPCR, normalized to CCR5 nuclear DNA, and plotted relative to the untreated lytic+casp-i sample. **B.** RNA was extracted and IFN-β mRNA levels were measured by RT-qPCR, normalized to 18S rRNA, and plotted relative to the lytic+casp-i sample. **C.** Cells were stained for KSHV ORF45 and analyzed by flow cytometry. ns = p>0.05, ** = p<0.01, **** = p<0.0001. Two-Way ANOVA followed by Tukey's post hoc multiple comparisons test for all experiments. n= 3 for all experiments.

Reviewer #2:
<u>Major comments</u>
Demonstrating coordinated occupancy or functional interplay of these factors would increase confidence in the proposed model and broaden the impact for readers interested in virology, immunology, and transcriptional regulation. […]
1) While the study presents intriguing evidence for AP-1 involvement in regulating IFN-β responses, the reliance on total c-Jun levels as a readout is limiting. Because c-Jun activity depends on phosphorylation and promoter binding, additional experiments (i.e., phospho-c-Jun analysis or ChIP at the IFNB1 promoter) would strengthen the link between AP-1 activity and the observed reporter outcomes.

We have analyzed the phosphorylation states of c-Jun and ATF2 and found that they are not different after lytic reactivation combined with caspase inhibition, and thus also between the IFN-β high and low cells. These results suggest that phosphorylation of these transcription factors is not occurring in this context. We have included these data as S5A-C Fig and mention them in the Results (lines 399-402) and Discussion sections (line 470-473). While we agree ChIP would be incredibly informative, it is unfortunately not feasible with the limiting number of IFN-β high cells that are obtainable from our FACS experiments. We have updated the Discussion section to explain this limitation (lines 483-487).

S5 Fig

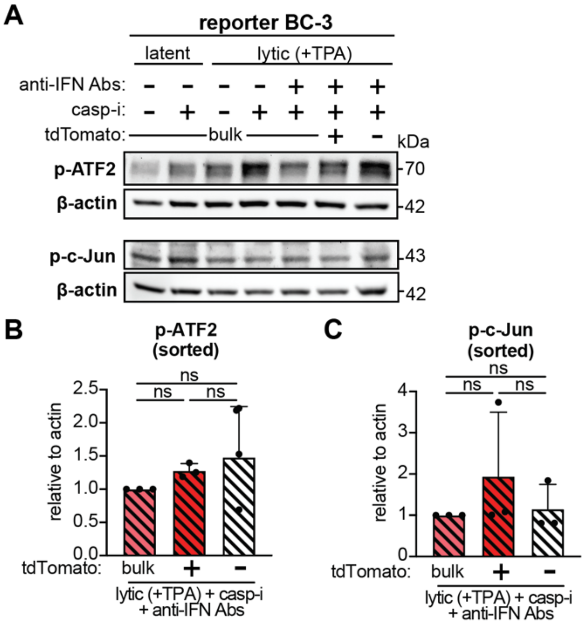

S5A-C Fig. AP-1 and NF-κB may be more active in IFN-β high cells. BC3-IFN-βp-tdTomato reporter cells were treated with 20 ng/ml TPA for 48 hours to induce the lytic cycle ("lytic (+TPA)"), 10 µM of the pan-caspase inhibitor IDN-6556 ("casp-i"), and a cocktail mix of antibodies against type I IFNs and their receptor ("anti-IFN Abs") to block IFN-β paracrine signaling. Protein was isolated from BC3-IFN-βp-tdTomato reporter cells without sorting ("bulk"), or after sorting the lytic+casp-i+anti-IFN Abs based on tdTomato expression. Western blots were probed for phosphorylated ATF2, phosphorylated c-Jun, and β-actin as a loading control. Levels of phosphorylated ATF2 (**B**) and phosphorylated c-Jun (**C**) in sorted samples were quantified from the western blots, normalized to β-actin, and plotted relative to bulk lytic+casp-i+ anti-IFN Abs samples. ns = p>0.05, * = p<0.05, ** = p<0.01. One-Way ANOVA followed by Tukey's post hoc multiple comparisons test for all experiments. n= 3 for A-C.

As suggested by reviewer 3, we have also carried out other experiments that speak to the involvement of the AP-1 complex. Using an AP-1/NF-κB inhibitor (S4A-B Fig) and mutating AP-1 and NF-κB binding sites in our reporter constructs (S4C-E Fig.) we now demonstrated that AP-1 and NF-κB complexes are needed for IFN induction in our system, both from the endogenous promoter and from the minimal promoter in the reporter constructs (Results section, lines 355-364).

S4 Fig

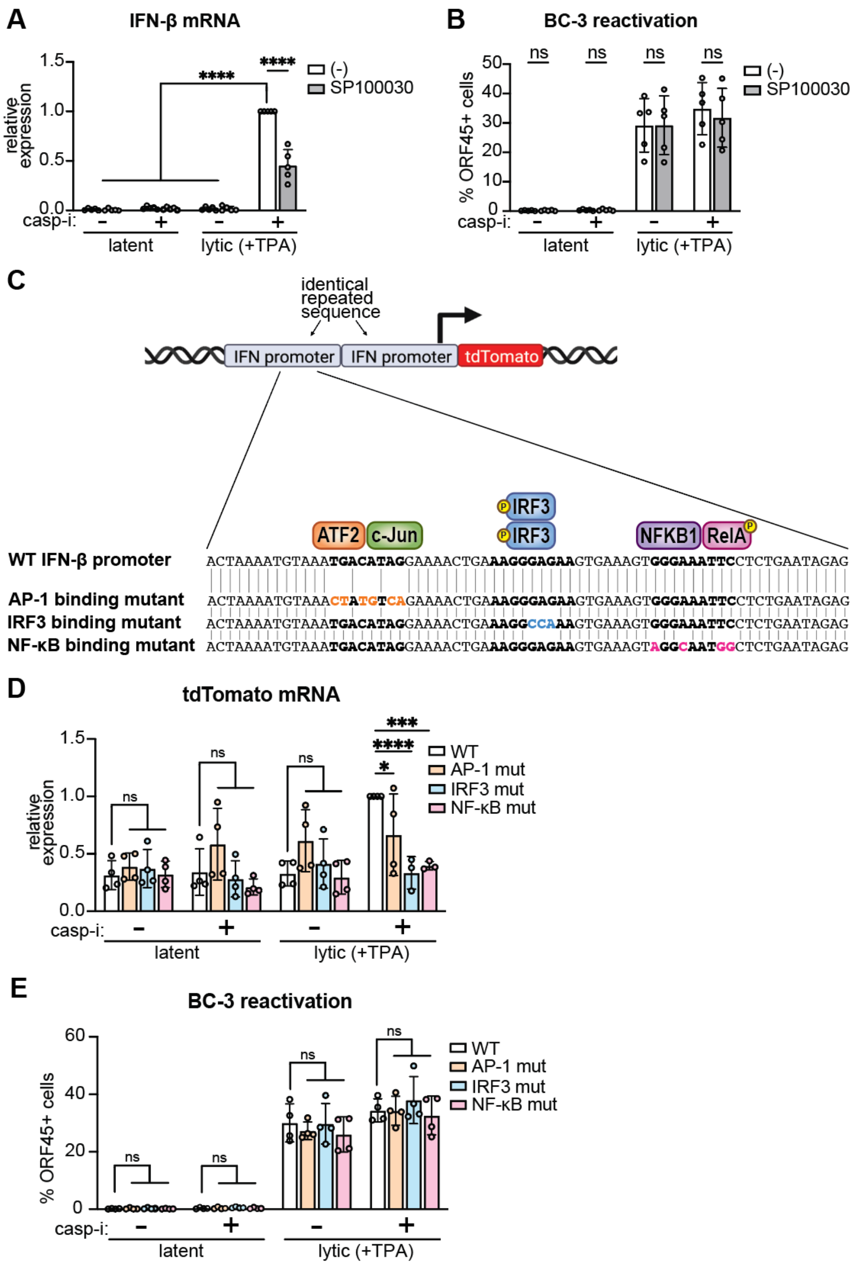

S4 Fig. IFN-β induction during KSHV lytic reactivation requires all three IFN-β enhanceosome transcription factors. A-B. BC-3 cells were treated with 20 ng/ml TPA for 48 hours to induce the lytic cycle (“lytic (+TPA)”), 10 μM of the pan-caspase inhibitor IDN-6556 (“casp-i”), and where indicated, 1 μM of the dual AP-1 and NF-κB inhibitor SP100030. **A.** RNA was extracted and IFN-β mRNA levels were measured by RT-qPCR, normalized to 18S rRNA, and plotted relative to the sample treated with TPA, casp-i, and vehicle control (“(-)”). **B.** Cells were stained for KSHV ORF45 and analyzed by flow cytometry. **C.** Diagram of the IFN-β promoter used in the reporter constructs, including mutations inserted to generate enhanceosome component binding mutants. Cassettes containing these mutant promoters were introduced in BC-3 cells via lentiviral transduction for stable integration. **D-E.** BC-3 cells with stably integrated tdTomato reporters driven by the promoters shown in **C** were treated with 20 ng/ml TPA for 48 hours to induce the lytic cycle (“lytic (+TPA)”) and 10 μM of the pan-caspase inhibitor IDN-6556 (“casp-i”). **D.** RNA was extracted and tdTomato mRNA levels were measured by RT-qPCR and normalized to 18S rRNA. mRNA levels are plotted relative to the cells containing the WT promoter-driven reporter, lytic+casp-i sample. **E.** Cells were stained for KSHV ORF45 and analyzed by flow cytometry. ns = p>0.05, *** = p<0.001, **** = p<0.0001. Two-Way ANOVA followed by Dunnett’s post hoc multiple comparisons test for all experiments. n= 5 for A-B, n= 4 for D-E

2) The data presented demonstrating that Serine 386 phosphorylation does not distinguish first responder cells is strong. Including complementary data on Ser396 phosphorylation would strengthen the conclusion, as this well-established activation marker is readily detectable with available reagents and would help confirm that the potentiation of IRF3 activity is not the driver of the observed heterogeneity.

We have performed experiments probing for IRF3 phosphorylation at the Serine 396 position and found the same pattern as for Serine 386. IRF3 serine 396 phosphorylation is increased only in lytic cells treated with caspase inhibitors and is detected in both IFN-β high and IFN-β low cells after sorting for tdTomato expression. We agree that these new results strengthen our findings and have added them as Fig 4F-G and K-L, and mentioned this in the text (lines 328-329).

Fig 4

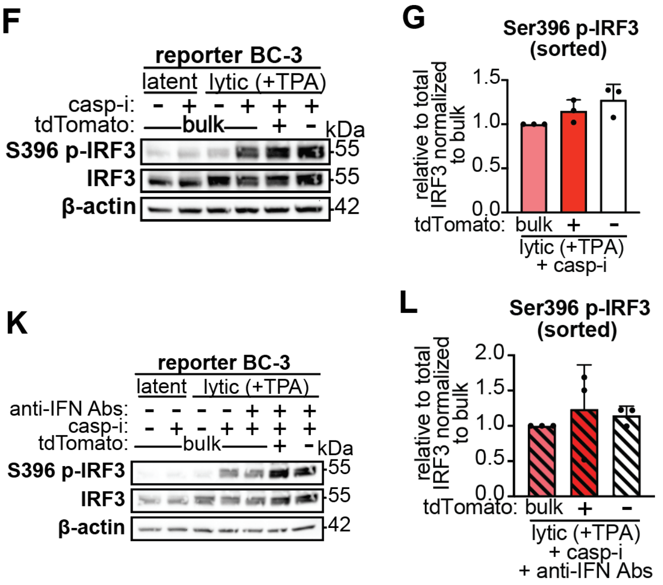

Fig 4 F-G, K-L. TBK1 and IRF3 are necessary but not sufficient to direct IFN-β expression, as they are activated even in cells with low IFN-β expression. BC3-IFN-βp-tdTomato reporter cells were treated with 20 ng/ml TPA for 48 hours to induce the lytic cycle ("lytic (+TPA)"). Where indicated, the cells were also treated with 10 μM of the pan-caspase inhibitor IDN-6556 ("casp-i"), and/or a cocktail of antibodies against type I IFNs and their receptor ("anti-IFN Abs") at 1:2000 dilution. Protein lysates were probed for phosphorylated IRF3 (Ser396), total IRF3, and β-actin as a loading control. Protein was isolated from treated BC3-IFN-βp-tdTomato reporter cells without sorting ("bulk"), or after sorting the lytic+casp-i (**F-G**) or lytic+casp-i+anti-IFN Abs (**K-L**) sample based on tdTomato expression. Western blot for phosphorylated IRF3 for bulk lytic+casp-i or lytic+casp-i+anti-IFN Abs treated samples and sorted tdTomato+ and tdTomato- samples were quantified by normalizing the density of phosphorylated protein to unphosphorylated protein bands. Phosphorylation levels are plotted relative to the bulk lytic+casp-i or lytic+casp-i+anti-IFN Abs treated samples.

Minor comments

3) Consider updating the title to more directly reflect the findings (e.g. "Interferon-β induction heterogeneity during KSHV infection is correlated to expression of ATF2 and RelA".

We have updated the title.

Previous title: Interferon-β induction heterogeneity during KSHV infection is correlated to expression and activation of enhanceosome transcription factors other than IRF3

Updated title: Interferon-β induction heterogeneity during KSHV infection is correlated to levels and activation of the transcription factors ATF2 and RelA, and not IRF3

4) To ease the interpretation of data, indicate what the black and white circles indicate in the figure legends.

We have updated all figures to have +/- symbols in place of the black and white circles to be more intuitive.

5) The authors predominantly rely on the IE gene ORF50 as a marker of KSHV reactivation and show no differences in expression between first responder cells and those that don't. Measurement of early and late genes across TdTomato-positive and negative cells would rule out potential differences in progression through reactivation that might influence IFN production.

We have now measured levels of additional KSHV genes, belonging to different kinetic classes (immediate early, early and late) in IFN-β high and low cells in the presence and absence of the paracrine signaling block (anti-IFN antibodies). We see no consistent differences between the IFN-β high and low cells. However, there is a trend of de-enrichment of late proteins, which agrees with our previous analysis that only cells in the early phase of the cycle induce IFN-β, as we discussed in the Introduction. We have added these data as S1 Fig and mention them in the text (lines 196-205).

S1 Fig

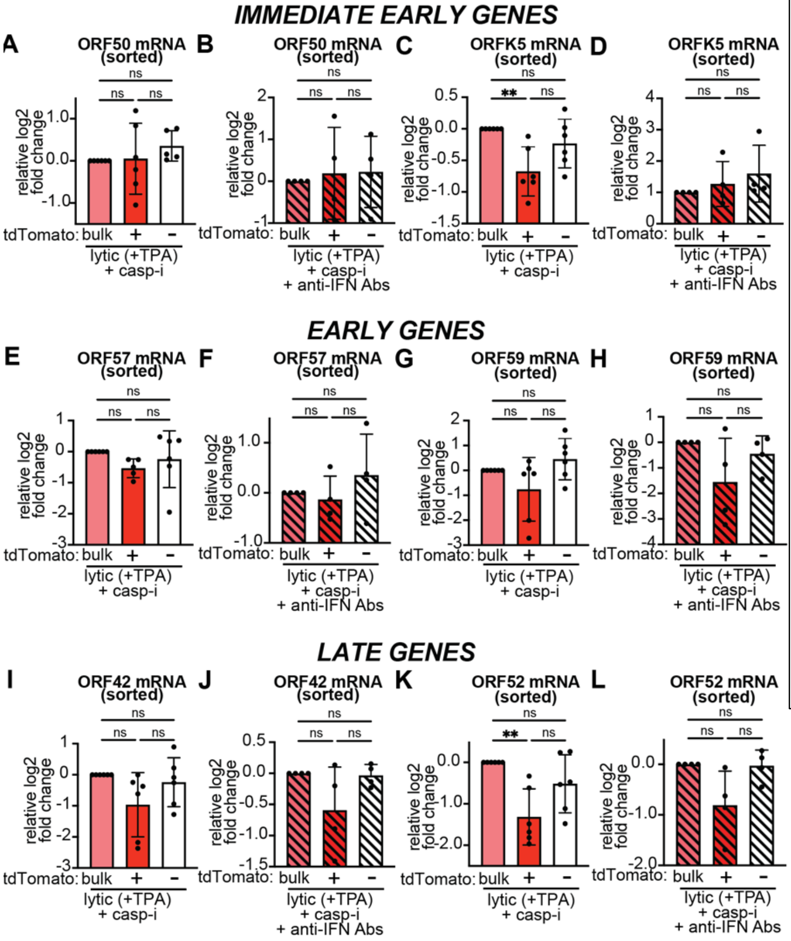

S1 Fig. Viral gene expression heterogeneity does not completely explain IFN-β expression. BC3-IFN-βp-tdTomato reporter cells were treated with 20 ng/ml TPA for 48 hours to induce the lytic cycle ("lytic (+TPA)"), 10 µM of the pan-caspase inhibitor IDN-6556 ("casp-i"), and/or a cocktail mix of antibodies against type I IFNs and their receptor ("anti-IFN Abs") at 1:2000 dilution. Cells from the lytic+casp-i (**A, C, E, G, I, K**) or the lytic+casp-i+anti-IFN Abs (**B, D, F, H, J, L**) samples were collected both without sorting ("bulk") and after sorting based on tdTomato expression (tdTomato + and -). RNA was extracted and KSHV mRNA levels were measured by RT-qPCR and normalized to 18S rRNA. Immediate early genes: ORF50 (**A-B**), ORFK5 (**C-D**). Early genes: ORF57 (**E-F**), ORF59 (**G-H**). Late genes: ORF42 (**I-J**), and ORF52 (**K-L**). The fold enrichment of mRNA levels compared to unsorted ("bulk") samples are plotted in log_2 scale. Results in this figure were obtained from the same samples used in Figs. 1 and 2 and are thus directly comparable to IFN-β enrichment shown in those Figure. ns = p>0.05, ** = p<0.01. One-Way ANOVA followed by Tukey's post hoc multiple comparisons test. n≥ 4 for all experiments.

6) The data in Figure 5D (quantified in E and F) show a compelling trend. This could be further clarified by plotting a trend line that connects the results of independent experiments, rather than only showing individual data points. Such visualization would make the consistency of the observed trend across experiments more apparent.

We have updated Fig 5E, F, and I to capture the mentioned trends by connecting each biological replicate with lines. We agree that this highlights the consistency of the observed trends by more clearly visualizing differences in ATF2 and p-RelA levels.

Fig 5

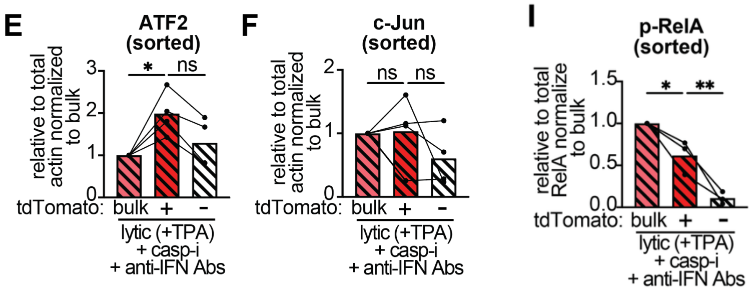

Fig 5E-F, I. Levels of the AP-1 subunit ATF2 and phosphorylation of the NF-κB subunit RelA are higher in cells with high IFN-β expression. E-F, I. BC3-IFN-βp-tdTomato reporter cells were treated with 20 ng/ml TPA for 48 hours to induce the lytic cycle (“lytic (+TPA)”), 10 µM of the pan-caspase inhibitor IDN-6556 (“casp-i”), and where indicated, a cocktail mix of antibodies against type I IFNs and their receptor (“anti-IFN Abs”) to block IFN-β paracrine signaling. Protein was isolated from BC3-IFN-βp-tdTomato reporter cells without sorting (“bulk”), or after sorting the lytic+casp-i+anti-IFN Abs based on tdTomato expression. Western blots were probed for ATF2, c-Jun, total RelA, phosphorylated RelA (Ser536), and β-actin as a loading control. Levels of ATF2 (**E**) and c-Jun (**F**) in sorted samples were quantified from the western blots, normalized to β-actin, and plotted relative to bulk lytic+casp-i+ anti-IFN Abs samples. Levels of phosphorylated RelA (Ser536) (**I**) were quantified by normalizing to total RelA and plotted relative to bulk lytic+casp-i+ anti-IFN Abs samples. ns = $p>0.05$, * = $p<0.05$, ** = $p<0.01$, One-Way ANOVA followed by Tukey’s post hoc multiple comparisons test.

Reviewer #3:

<u>Major comment</u>

a major worry comes from using lentiviral transduction to insert the reporter promoter into cells without selecting for clones. Lentiviral transduction introduces heterogeneity due to random insertion of their vector. This results in different copy numbers for the reporter construct, leading to heterogeneity in the reporter expression. Additionally, the expression of foreign proteins, particularly in immune cells, can be perceived as danger signals (10.1007/s12015-016-9670-8) and occasionally trigger p65 activation. To control for this, the authors could validate their reporter results by including a non-IFNb promoter (e.g., constitutive) expressing tdTomato and verifying that these cell populations do not also express endogenous IFNb mRNAs.

To address this concern and test for heterogeneity effects from lentiviral transduction, we generated a BC-3 line where tdTomato expression is driven by a CMV promoter instead of the IFN-β promoter. When we sorted tdTomato-expressing cells from this line, we found that there was no enrichment for IFN-β expression, indicating that heterogenous reporter insertion and/or reporter expression alone do not drive IFN-β expression. We have included these data as S2 Fig, and updated the Results sections (line 206-217) to mention this.

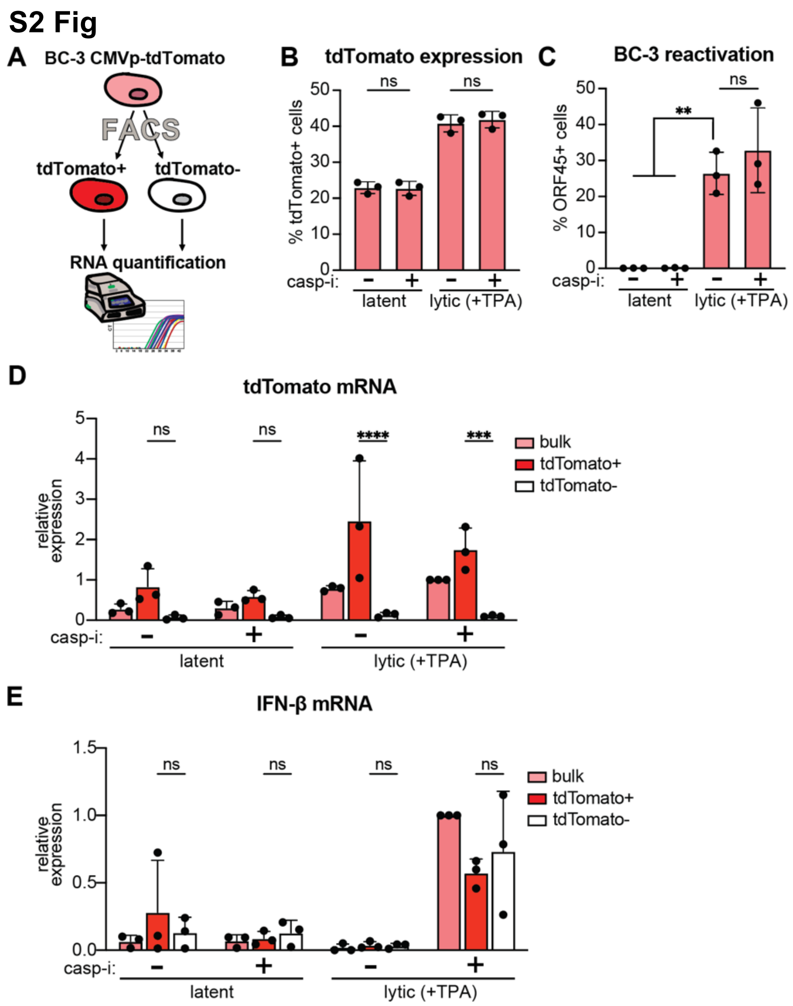

S2 Figure. Lentivirally established CMV promoter-driven tdTomato expression does not induce IFN-β in BC-3 cells and does not correlate with IFN-β expression. A. Diagram of CMV promoter-driven tdTomato sorting strategy. A cassette containing the tdTomato fluorophore driven by a CMV promoter was introduced in BC-3 cells via lentiviral transduction for stable integration. Cells were sorted based on tdTomato expression and RNA samples were collected from the same experiment. **B-E.** BC3-CMVp-tdTomato cells were treated with 20 ng/mL TPA for 48 hours to induce the lytic cycle ("lytic (+TPA)"). Where indicated, the cells were also treated with 10 μM of the pan-caspase inhibitor IDN-6556 ("casp-i"). **B.** Cells were analyzed by flow cytometry for tdTomato fluorescence. **C.** Cells were stained for KSHV ORF45 and analyzed by flow cytometry. **D-E.** RNA was isolated from cells collected without sorting ("bulk") or after sorting based on tdTomato expression (tdTomato+ and -). IFN-β and tdTomato mRNA levels were measured by RT-qPCR and normalized to 18S rRNA. mRNA levels are plotted relative to the lytic+casp-i bulk sample. ns = p>0.05, ** = p<0.01, *** = p<0.001, **** = p<0.0001. One-Way ANOVA followed by Tukey's (B) or Dunnett's (C) post hoc multiple comparisons test. Two-Way ANOVA followed by Tukey's post hoc multiple comparisons test (D, E). n= 3 for all experiments.

We have also included new experiments using reporters containing mutations in the transcription factor binding sites in the IFN-β promoter to ensure that our reporter faithfully reports on IFN-β induction regardless of lentiviral transduction. We found that mutating any of the IFN-β enhanceosome binding sites prevents tdTomato induction (S4C-E Fig, Results section lines 359-364). We also updated the text in the Results section to mention that our lack of clonal selection was intentional, as we did not want the tdTomato signal to reflect specific regulation of a single genomic region (lines 165-167).

S4 Fig

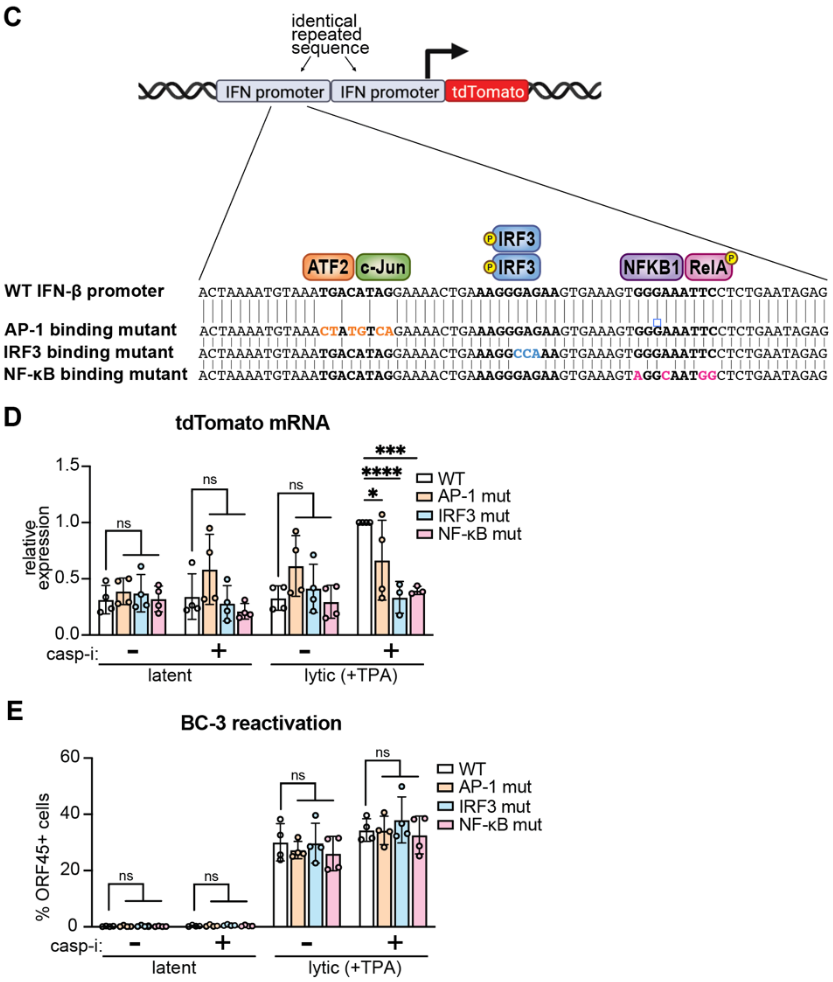

S4C-E Fig. IFN-β induction during KSHV lytic reactivation requires all three IFN-β enhanceosome transcription factors. C. Diagram of the IFN-β promoter used in the reporter constructs, including mutations inserted to generate enhanceosome component binding mutants. Cassettes containing these mutant promoters were introduced in BC-3 cells via lentiviral transduction for stable integration. **D-E.** BC-3 cells with stably integrated tdTomato reporters driven by the promoters shown in **C** were treated with 20 ng/ml TPA for 48 hours to induce the lytic cycle ("lytic (+TPA)") and 10 μM of the pan-caspase inhibitor IDN-6556 ("casp-i"). **D.** RNA was extracted and tdTomato mRNA levels were measured by RT-qPCR and normalized to 18S rRNA. mRNA levels are plotted relative to the cells containing the WT promoter-driven reporter, lytic+casp-i sample. **E.** Cells were stained for KSHV ORF45 and analyzed by flow cytometry. ns = p>0.05, *** = p<0.001, **** = p<0.0001. Two-Way ANOVA followed by Dunnett's post hoc multiple comparisons test for all experiments. n= 4 for D-E.

Minor comments

Regarding AP1 and NF-kB activation, the authors could investigate downstream genes such as GADD45B, HSPA1A, and ATF-3 (for AP1), and IL-6, TNFAIP3 (A20) (for both AP1and NFkB). It would be interesting to determine if these genes are exclusively expressed in tdTomato-expressing cells.

We have now measured levels of GADD45B, HSPA1A, ATF-3, IL-6, and TNFAIP3 in IFN-β high and low cells in the presence of the paracrine signaling block (anti-IFN antibodies). We observed enrichment of ATF-3 and TNAIP3 in the tdTomato-positive IFN-β high cells. We agree that this strengthens our findings by demonstrating AP-1 and NF-κB activity in these cells, and have included these data as S5D-E Fig and added them to the Results section (line 409-413).

S5 Fig

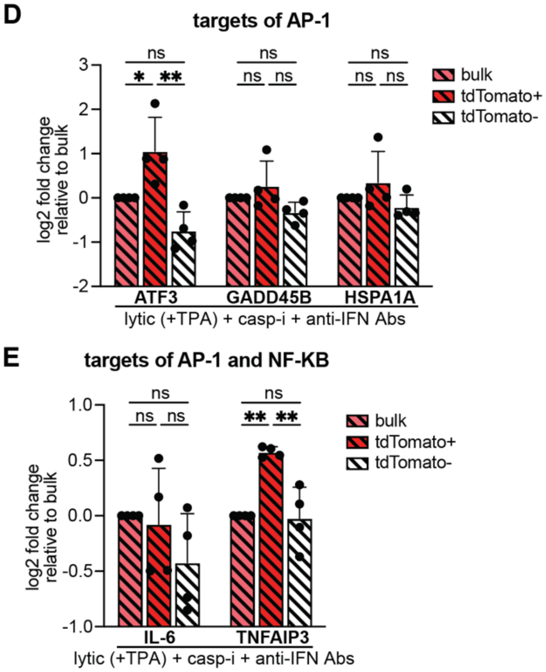

S5D-E Fig. AP-1 and NF-κB may be more active in IFN-β high cells. BC3-IFN-βp-tdTomato reporter cells were treated with 20 ng/ml TPA for 48 hours to induce the lytic cycle ("lytic (+TPA)"), 10 μM of the pan-caspase inhibitor IDN-6556 ("casp-i"), and a cocktail mix of antibodies against type I IFNs and their receptor ("anti-IFN Abs") to block IFN-β paracrine signaling. RNA was extracted from BC3-IFN-βp-tdTomato reporter cells without sorting ("bulk"), or after sorting the lytic+casp-i+anti-IFN Abs based on tdTomato expression (tdTomato+ and -). mRNA levels of targets of AP-1 – ATF3, GADD45B, and HSPA1A (**D**) – and targets of AP-1 and NF-κB – IL-6 and TNFAIP3 (**E**) – were measured by RT-qPCR, normalized to 18S rRNA, and plotted relative to the bulk lytic+casp-i+anti-IFN Abs sample. ns = p>0.05, * = p<0.05, ** = p<0.01. One-Way ANOVA followed by Tukey's post hoc multiple comparisons test for all experiments. n= 4 for D-E.

While the authors observed no direct correlation between c-Jun alone and IFN-b production, it is conceivable that TPA-induced c-Jun primes the cells that become fully transcriptionally active upon a stimulus like viral reactivation. I propose that the authors attempt to inhibit c-Jun activation during KSHV reactivation (TPA + caspase inhibitor) using inhibitors like SP600125 and subsequently assess whether this blockade reduces the proportion of IFNb+ cells.

We tried using SP600125 to inhibit JNK signaling, but this drug significantly inhibited KSHV reactivation, making it difficult to discern whether impacts on IFN-β induction are due to inhibited reactivation or JNK signaling. Instead, we used SP100030, a dual AP-1 and NF-κB inhibitor. We show that one or both of these factors are needed for IFN-β induction without impact on viral reactivation. We have included these data in S4A-B Fig and text of Results section (line 355-359).

S4 Fig

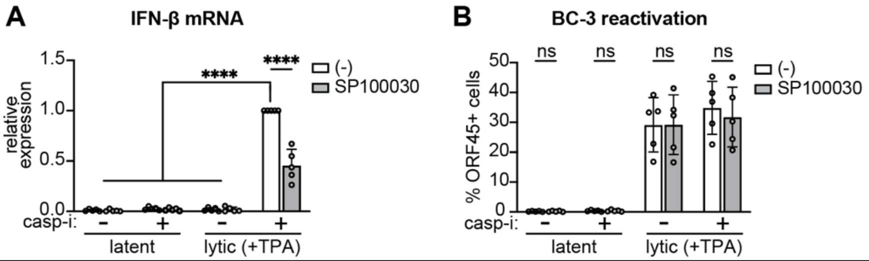

S4A-B Fig. IFN-β induction during KSHV lytic reactivation requires all three IFN-β enhanceosome transcription factors. BC-3 cells were treated with 20 ng/ml TPA for 48 hours to induce the lytic cycle ("lytic (+TPA)"), 10 μM of the pan-caspase inhibitor IDN-6556 ("casp-i"), and where indicated, 1 μM of the dual AP-1 and NF-κB inhibitor SP100030. **A.** RNA was extracted and IFN-β mRNA levels were measured by RT-qPCR, normalized to 18S rRNA, and plotted relative to the sample treated with TPA, casp-i, and vehicle control ("(-)"). **B.** Cells were stained for KSHV ORF45 and analyzed by flow cytometry. ns = $p>0.05$, *** = $p<0.001$, **** = $p<0.0001$. Two-Way ANOVA followed by Dunnett's post hoc multiple comparisons test for all experiments. n= 5 for A-B
